# Prevalence of *Staphylococcus* spp. nasal colonization among doctors of podiatric medicine and associated risk factors in Spain

**DOI:** 10.1186/s13756-018-0318-0

**Published:** 2018-02-17

**Authors:** Sheila de Benito, Luis Alou, Ricardo Becerro-de-Bengoa-Vallejo, Marta Elena Losa-Iglesias, María Luisa Gómez-Lus, Luis Collado, David Sevillano

**Affiliations:** 1 Facultad de Ciencias de la Salud, Universidad Rey Juan Carlos, Madrid, Spain; 2 Area de Microbiología, Facultad de Medicina, Universidad Complutense de Madrid, Madrid, Spain; 3 Facultad de Enfermería, Fisioterapia y Podología, Universidad Complutense de Madrid, Madrid, Spain; 4 Departamento de Medicina, Facultad de Medicina, Universidad Complutense de Madrid, Madrid, Spain

**Keywords:** *Staphylococcus aureus*, *Staphylococcus epidermidis*, Nasal carriage, Podiatrists, Methicillin

## Abstract

**Background:**

This study aimed to estimate the prevalence of methicillin-susceptible and -resistant *Staphylococcus aureus* (MSSA and MRSA) and methicillin-resistant *Staphylococcus epidermidis* (MRSE) nasopharyngeal carriage among Doctors of Podiatric Medicine (Podiatrists) and to determine the potential risk factors.

**Methods:**

A cross-sectional study was carried out in 2016–2017 among 239 podiatrists in Spain. The presence of MSSA, MRSA, and MRSE was determined by microbiological analysis of nasal exudate and antimicrobial susceptibility was determined. Each podiatrist completed a questionnaire. The questionnaire comprised various parameters such as sex, age, podiatry experience duration, underlying diseases, prior antibiotic treatment, hospitalization during the last year, and use of a protective mask, an aspiration system, or gloves.

**Results:**

The prevalence of MSSA, MRSA, and MRSE was 23.0%, 1.3%, and 23.8%, respectively. The MSSA prevalence was higher among podiatrists who did not use an aspiration system (32.3%) compared to those who did (19.3%; *p* = 0.0305), and among podiatrists with respiratory diseases (36.8%) compared to those without (20.8%; *p* = 0.0272). The MRSE prevalence was higher among men (33.7%) compared to women (8.6%; *p* = 0.0089), podiatrists aged ≥50 (38.5%) compared to ≤35 (17.8%; *p* = 0.0101), and podiatrists with ≥15 (39.3%) compared to ≤5 years of podiatry experience (12.5%; *p* = 0.0015). Among the *S. aureus* strains, 84.5% were resistant to penicillin, 22.4% to erythromycin, 20.7% to clindamycin, and 12.7% to mupirocin. The MRSE strains were resistant to penicillin (93.0%), erythromycin (78.9%), and mupirocin (73.7%).

**Conclusions:**

The prevalence of *S. aureus* and *S. epidermidis* nasal carriage is low among Spanish podiatrists compared to other health professionals.

**Electronic supplementary material:**

The online version of this article (10.1186/s13756-018-0318-0) contains supplementary material, which is available to authorized users.

## Background

Humans are a natural reservoir of *Staphylococcus aureus* [1]. The nasal cavity is the main reservoir, but *S. aureus* can colonize other areas of the body such as the skin, perineal region, and pharynx [2]. Studies have shown that there are three types of nasal carriers among healthy individuals: 20% are persistent carriers (range 12–30%), 30% are intermittent carriers (16–70%), and 50% (16–69%) are non-carriers [2, 3].

The prevalence of *S. aureus* nasal carriage varies by country, profession, and demographic group. In the general population, values range from 17.8% to 21.6% in European countries [4, 5] to a prevalence of 31.6% in the US [6]. A higher prevalence of *S. aureus* among health professionals has been observed, with values of 52% among physiotherapists [7], 36–66% among nurses [8, 9], and 30.6% among pediatricians [10], but the prevalence in Doctors of Podiatric Medicine (Podiatrists) as members of the surgical and medical team has not been described yet in any country. There are also specific demographic groups that have higher percentages of nasal carriers. Risk factors are Caucasian ethnicity, male sex [11], advanced age [12] and use of hormonal contraceptives [13]. Additional factors include medical conditions such as insulin-dependent and -independent diabetes [14], patients undergoing hemodialysis [15], HIV [16], people with obesity [17] and patients with skin infections due to *S. aureus* or diseases of the skin [18].

Being a nasal carrier of *S. aureus* has been identified as a risk factor for the development of nosocomial and community-acquired staphylococcal infections, as it provides a reservoir from which bacteria can spread when the host’s defenses are compromised [19].

*S. aureus* is one of the most common and clinically significant pathogens, causing a broad spectrum of nosocomial and community-acquired infection. It has the capacity to cause skin and soft tissue infections, osteoarticular infections, and serious systemic infections such as bacteremia and endocarditis. *S. aureus* is the second most common cause of bacteremia and the most common cause of nosocomial bacteremia in Europe [20].

The variability of *S. aureus*, its rapid adaptive response to environmental changes, and its continuous acquisition of antibiotic resistance factors have made it a common resident in hospitals, where it causes multiresistance problems, which can occasionally be considerable. Although the term “methicillin resistance” implies resistance to β-lactam derivatives, methicillin-resistant *S. aureus* (MRSA) strains generally also have resistance to other groups of antibiotics. Via several mechanisms, these isolates can have resistance to chloramphenicol, tetracyclines, macrolides, lincosamines, aminoglycosides, and even quinolones, with MRSA outbreaks being increasingly reported [21].

*S. epidermidis* used to be considered a commensal microorganism. However, nowadays, it is considered an important opportunistic pathogen, producing a great variety of infections of varying severity and acting as a significant agent in medical implant infections. In addition, it is considered a potential reservoir of resistance genes for pathogenic bacteria, such as *S. aureus*, increasing the potential of *S. aureus* to colonize, survive during infection, and resist antibiotic treatment, which are important features of MRSA [22].

The objective of this study was to estimate the prevalence of nasal carriage of methicillin-susceptible and -resistant *S. aureus* (MSSA and MRSA) and methicillin-resistant *S. epidermidis* (MRSE) among podiatrists in Spain (Europe), to identify the possible risk factors for colonization of both bacteria in this population, and to determine the levels of antibiotic susceptibility among the isolates.

## Methods

The study was a cross-sectional study according to the STROBE statement involving 239 active podiatrists from all the states of Spain. Nasal exudate sample collection and processing were carried out between September 2016 and February 2017 in the Microbiology Department of the Complutense University of Madrid. The samples were collected after the podiatrists signed an informed consent form and completed a supplemental questionnaire.

The dichotomous dependent variables studied were the *S. aureus* and MRSE carriage status, as well as the status of co-colonization by both bacteria. The independent variables were sex, age, years of podiatry experience, underlying disease (diabetes, respiratory disease, immunosuppressive disease, and infectious disease), antibiotic therapy prior to sampling, hospitalization during the previous year, and use of a respiration mask, aspiration system, and gloves.

### Sample size

The sample size was calculated with software from Unidad de Epidemiología Clínica y Bioestadística, Complexo Hospitalario Universitario de A Coruña, Universidade A Coruña (www.fisterra.com). The calculations were based on the total population living in Spain, which amounted to 42,104,557 adults on January 1, 2017. (http://www.ine.es). It was determined that, based a desired power of 80% with a β level of 20%, and a precision of 3% with an α level of 0.05, with a confidence interval of 95%, for a proportion of 50%, assuming a loss of 15%, at least 239 participants were included in the study.

### Sample collection, processing, and antimicrobial testing

Samples were obtained from the front of the nostrils of each individual using a sterile swab, rotating it gently in one of the nasal cavities at least five times. The nasal swabs were streaked in two chromogenic culture media. The first was selective for the isolation of strains in the *Staphylococcus* genus and differential for *S. aureus*, i.e., CHROMagar Staph aureus medium (Becton Dickinson, Sparks, MD), and the second was for the identification of MRSA, i.e., CHROMagar MRSA medium (Becton Dickinson, Sparks, MD) with methicillin. If colonies were observed in the culture media, we used biochemical tests (mannitol agar, coagulase, polymixin B disc, and pyrrolidonyl aminopeptidase test (Becton Dickinson, Sparks, MD) to confirm and identify the strains.

An antimicrobial susceptibility analysis was performed using the agar disk-diffusion method, following the indications and interpretation criteria of the Clinical & Laboratory Standards Institute [23]. The antibiotics used to perform the antibiogram were clindamycin (2 μg), erythromycin (15 μg), penicillin (10 μg), chloramphenicol (30 μg), vancomycin (30 μg), mupirocin (5 μg), rifampicin (5 μg), tetracycline (30 μg), ciprofloxacin (5 μg), gentamicin (10 μg), cefoxitin (30 μg), linezolid (30 μg), and trimethoprim/sulfamethoxazole (23.75/1.25 μg) (cotrimoxazole). Erythromycin and clindamycin discs were assessed together to detect inducible macrolide-lincosamide streptogramin B (iMLS_B_) phenotype.

### Statistical analysis

We used the non-parametric Mann–Whitney U test or the independent t-test to establish whether there were differences between groups. The chi square test was used to analyze the categorical variables. The data was analyzed using SPSS version 19 (IBM Corp., Armonk, NY, US). The level of significance was set at *p* < 0.05.

## Results

A total of 239 samples were analyzed. The mean age was 33.3 ± 8.5 years; 65% (115/239) of the subjects were women; and the mean number of years of podiatry experience was 9.9 ± 7.7.

The prevalence of *S. aureus* carriers among the podiatrists was 24.3% (58/239), of which 23% (55/239) were MSSA carriers and 1.3% (3/239) were MRSA carriers.

Table 1 shows the participants’ characteristics and the results of the independent analyses of *S. aureus* and *S. epidermidis* carriers.

**Table 1:** Results of the analysis of *S. aureus* and *S. epidermidis* carriers among podiatrists

Variable	Category	N total (%)	*S. aureus*						*S. epidermidis*		
			MSSA		P	MRSA		P	MRSE		P
			N	%		N	%		N	%	
SEX	Female	156 (65)	30	19.2		2	1.2		29	8.6	
	Male	83 (35)	25	30.1	0.0569	1	1.3	0.9593	28	33.7	0.0089
AGE	≤35	157 (65.7)	33	21.0		1	0.7		28	17.8	
	36 to 49	69 (28.9)	17	24.6		2	3.9		24	34.7	
	≥50	13 (5.4)	5	38.5	0.3318	0	0.0	0.3408	5	38.5	0.0101
Years of Podiatry Experience	≤ 5	80 (33.5)	17	21.3		1	1.2		10	12.5	
	6 to 14	103 (43.1)	21	20.3		1	1.0		25	24.2	
	≥ 15	56 (23.4)	17	29.8	0.3254	1	1.8	0.9074	22	39.3	0.0015
Use of a protection mask	Yes	216 (90.4)	51	23.6		3	1.4		53	24.5	
	No	23 (9.6)	4	17.4	0.5005	0	0.0	0.5695	4	17.4	0.4446
Use of gloves	Yes	237 (99.1)	55	23.2		3	1.3		57	24.0	
	No	2 (0.8)	0	0.0	0.4375	0	0.0	0.8728	0	0.0	0.4267
Use of an aspiration system	Yes	171 (71.5)	33	19.3		2	1.2		44	25.7	
	No	68 (28.5)	22	32.3	0.0305	1	1.5	0.8504	13	19.1	0.2791
Concomitant disease	a	187 (78)	39	20.8	0.1330	3	1.6	0.3580	51	27.3	0.0185
	b	0 (0.0)	0	0.0	─	0	0.0	─	0	0.0	─
	c	38 (16)	14	36.8	0.0272	0	0.0	0.4485	5	13.2	0.1795
	d	1 (0.4)	0	0.0	─	0	0.0	─	0	0.0	─
	e	9 (3.7)	2	22.2	0.9542	0	0.0	0.0870	1	11.0	0.3607
	b,d	2 (0.8)	0	0.0	─	0	0.0	─	0	0.0	─
	b,e	1 (0.4)	0	0.0	─	0	0.0	─	0	0.0	─
	d,e	1 (0.4)	0	0.0	─	0	0.0	─	0	0.0	─

ABREVIATIONS: (a) no disease; (b) diabetes; (c) respiratory disease; (d) immunosuppresive disease; (e) infectious disease

Although no significant differences were observed, MSSA was found to be more common in men (30.1%; 25/83) than women (19.2%; 33/171). In addition, it was more common in older subjects, with a 21% (33/157) prevalence among podiatrists aged ≤35 years vs. 38.5% (5/13) among podiatrists aged ≥50 years. Moreover, it was more common in subjects with more podiatry experience, with a 21.3% (17/80) prevalence among podiatrists with ≤5 years of experience vs. 29.8% (17/56) among podiatrists with ≥15 years of experience.

Regarding the use of protective measures, we found a higher prevalence of MSSA among podiatrists who did not use an aspiration system in their usual work practice (32.3%; 22/68) compared to those who did (19.3%; 33/171) (*p* = 0.0305). Regarding concomitant diseases, a significantly higher prevalence was observed among podiatrists with respiratory diseases (36.8%; 14/38) compared to those without respiratory diseases (20.8%; 39/187) (*p* = 0.0272). No significant differences in the prevalence of MSSA were found for any of the other diseases we examined (i.e., diabetes and immunosuppressive and infectious diseases).

Regarding MRSA, a similar prevalence was found for males (1.3%; 1/83) and females (1.2%; 2/156), and for participants with different podiatry experience, with a prevalence of 1.2% (1/80) among podiatrists with ≤5 years of experience compared to 1.8% (1/56) among those with ≥15 years of experience.

The overall prevalence of MRSE was 23.8% (57/239). Like *S. aureus*, the prevalence of MRSE was significantly greater in men (33.7%; 28/83) compared to women (8.6%; 29/156) (*p* = 0.0089). We observed a significantly higher prevalence among podiatrists aged ≥50 years (38.5%; 5/13) compared to those aged ≤35 years (17.8%; 28/157) (*p* = 0.0101). Regarding podiatry experience, a higher prevalence was found among podiatrists with ≥15 years of experience (39.3%; 22/56) compared to those with ≤5 years of experience (12.5%;10/80) (*p* = 0.0015). A significantly higher prevalence of MRSE was found among podiatrists who did not have any of the diseases studied (27.3%; 51/187) compared to those with a disease (11.5%; 6/52) (*p* = 0.0185).

In addition, 7.5% (18/239) of the podiatrists had a co-colonization by both bacteria: 7.1% (17/239) were carriers of MSSA and MRSE while 0.4% (1/239) had MRSA and MRSE. A significantly higher prevalence of co-colonization was found in men (15.7%; 13/83) than in women (3.2%; 5/156) (*p* = 0.0005). This was also the case for older (≥50 years) vs. younger (≤35 years) podiatrists, with a prevalence of 30.8% (4/13) and 3.8% (6/157), respectively (p = 0, 0006). Finally, the prevalence varied with podiatry experience, with those who had ≥15 years of experience having a higher prevalence (17.9%; 10/56) than those with ≤5 years of experience (2.5%; 2/80) (*p* = 0.0026). This is similar to what was found for independent colonization by each bacterium.

Tables 2 and 3 show the results of the antimicrobial susceptibility tests for *S. aureus* and *S. epidermidis*. We found that 84.5% (49/58) of *S. aureus* had resistance to penicillin, 22.4% (13/58) to erythromycin, and 20.7% (12/58) to clindamycin. A 7.2% (4/55) and 0% of MSSA and MRSA strains showed iMLS_B_ phenotype, respectively. In addition, 12.7% (7/55) of the strains were resistant to mupirocin. Only three *S. aureus* isolates (5.2%; 3/58) had resistance to methicillin (MRSA), and none were resistant to rifampicin, linezolid, gentamicin, tetracycline, or vancomycin.

**Table 2:** Antimicrobial susceptibility of isolated *S. aureus* strains

Antibiotic	Number of strains			%		
	S	I	R	S	I	R
RIFAMPICIN	58	0	0	100.0	0.0	0.0
CHLORAMPHENICOL	50	4	4	86.2	6.9	6.9
LINEZOLID	58	0	0	100.0	0.0	0.0
GENTAMICIN	58	0	0	100.0	0.0	0.0
PENICILLIN	9	0	49	15.5	0.0	84.5
COTRIMOXAZOLE	51	2	5	88.0	3.4	8.6
CEFOXITIN	55	0	3	94.8	0.0	5.2
TETRACYCLINE	58	0	0	100.0	0.0	0.0
VANCOMYCIN	58	0	0	100.0	0.0	0.0
CIPROFLOXACIN	54	4	0	93.1	6.9	0.0
ERYTHROMYCIN	45	0	13	77.6	0.0	22.4
CLINDAMYCIN	46	0	12	79.3	0.0	20.7
MUPIROCIN	51	0	7	88.0	0.0	12.0

ABBREVIATIONS: *S* susceptible, *I* intermediate susceptibility, *R* resistant

**Table 3:** Antimicrobial susceptibility of isolated MRSE strains

Antibiotic	Number of strains			%		
	S	I	R	S	I	R
RIFAMPICIN	55	0	2	96.5	0.0	3.5
CHLORAMPHENICOL	56	1	0	98.2	1.8	0.0
LINEZOLID	57	0	0	100.0	0.0	0.0
GENTAMICIN	46	0	11	80.7	0.0	19.3
PENICILLIN	4	0	53	7.0	0.0	93.0
COTRIMOXAZOLE	44	0	13	77.2	0.0	22.8
CEFOXITIN	0	0	57	0.0	0.0	100.0
TETRACYCLINE	42	0	15	73.7	0.0	26.3
VANCOMYCIN	57	0	0	100.0	0.0	0.0
CIPROFLOXACIN	39	2	16	68.4	3.5	28.1
ERITHROMYCIN	12	0	45	21.1	0.0	78.9
CLINDAMYCIN	36	0	21	63.2	0.0	36.8
MUPIROCIN	15	0	42	26.3	0.0	73.7

ABBREVIATIONS: *S* susceptible, *I* intermediate susceptibility, *R* resistant

Antimicrobial resistance rates among the MRSE strains were higher than among the *S. aureus* strains. The antibiotics with the highest level of resistance were penicillin (93.0%; 53/57), erythromycin (78.9%; 45/57), and mupirocin (73.7%; 42/57). A 36.8% (21/57) were resistant to clindamycin of which a 10.5% (6/57) showed iMLS_B_ phenotype. None of the MRSE strains were resistant to linezolid, chloramphenicol or vancomycin.

Table 4 shows the number of antibiotics to which stains of the two species were resistant. We found that 93.0% (53/57) of MRSE, 33.3% (1/3) of MRSA, and 20% (11/55) of MSSA strains were resistant to three or more antibiotics.

**Table 4:** Number of antibiotics to which each strain was resistant

Strain	Total N	Number of antibiotics to which the strains were resistant							
		0		1		2		≥3	
		N	%	N	%	N	%	N	%
MSSA	55	7	12.7	28	50.9	9	16.4	11	20.0
MRSA	3	0	0.0	1	33.3	1	33.3	1	33.3
MRSE	57	0	0.0	0	0	4	7.0	53	93.0

ABBREVIATIONS: *ATB* antibiotic, *0 ATB* resistant to zero antibiotics, *1 ATB* resistant to one antibiotic, *2 ATB* resistant to two antibiotics, *≥3 ATB* resistant to three or more antibiotics

## Discussion

The prevalence of nasal carriers of *S. aureus* varies greatly between different countries and health professional categories; for example, the prevalence of nasal carriers of *S. aureus* among different health personnel has been reported to vary between 22.7% to 48% [8–10]. However, in our study, we observed a prevalence of *S. aureus* among podiatrists of 24.3%, which falls within the prevalence range observed in general population-based studies (17.8–31.6%) [4, 6].

Regarding the prevalence of MRSA carriers among health workers, previous studies have reported that it varies between 1.5% and 8.7% [9, 10, 24, 25], which is higher than the prevalence detected in our study of podiatrists (1.3%). A review published in 2014 that included 31 studies of different health personnel estimated that there is a mean prevalence of MRSA carriers of 4.0% in Europe and 6.6% in the US [26]. In contrast, the prevalence of MRSA among the podiatrists in our study falls within the prevalence range observed in general population-based studies (0.7 to 2.1%) [5, 6, 27].

Regarding the possible risk factors, although the differences were not significant, the prevalence of *S. aureus* nasal carriage was higher in older clinicials and those with more years of podiatry experience. This shows that the longer the exposure to the infectious agent, the more likely one is to be colonized. There are numerous longitudinal studies that corroborate this observation [4, 28–30], such as a study conducted among medical students at the Complutense University of Madrid. This study found the prevalence of *S. aureus* and MRSA colonization to be 26.92% and 0%, respectively, for students in their third course of study. These values were higher 3 years later, when the students were in their 6th year (46.25% and 1.25%, respectively). A similar trend was reported by Güçlü et al., who found 13.4% of preclinical students to be colonized, and 42% of those in their 5th year of study. In addition, we also observed a higher prevalence among podiatrists who did not use an aspiration system, demonstrating that protective measures are important to avoid the spread of and/or colonization by bacteria.

The *S. aureus* nasal carrier status is epidemiologically related to the exacerbation of allergic conditions, especially allergic rhinitis. In our study, 34.1% of the podiatrists with respiratory diseases (such as rhinitis and asthma) were carriers of MSSA, and we found a significant difference with those who did not have respiratory diseases (*p* = 0.0272). This supports the possible association between the presence of such bacteria and allergies.

Regarding antimicrobial susceptibility, the *S. aureus* isolates showed resistance to penicillin (84.5%), erythromycin (22.4%), and clindamycin (20.7%), which is similar to the findings of other recent studies [5, 9, 31]. In our study, 20.6% of the *S. aureus* isolates had multiresistance (resistance to three or more antibiotics). This is higher than the values reported in previous studies, in which values range from 0.2% to 7.1% [5, 32].

Among the podiatrists, the prevalence of MRSE was 23.8%, which is similar to the results of a previous study that investigated the prevalence of coagulase-negative *Staphylococcus* (CoNS) [30] but much higher than the prevalence of *S. epidermidis* reported in students in Vienna, Austria that were 2.5% [33].

The levels of antibiotic resistance among the MRSE isolates observed in our study are very like those reported in other studies, except for the levels of resistance to erythromycin and mupirocin (78.9% and 73.7%, respectively), which were higher than those found in a previous study in Spain [30]. Although mupirocin is the antibiotic of choice for the eradication of *Staphylococcus* colonization, resistance to this antibiotic has increased in recent years [34, 35]. CoNS has higher levels of resistance to mupirocin than *S. aureus* [36]. These data raise the question of whether the risk of acquisition of mupirocin resistance by *S. aureus* isolates increases in cases of co-colonization with mupirocin-resistant CoNS, especially when *S. aureus* is under selective pressure due to mupirocin administration [37]. Lastly, we found that 93% of the MRSE strains had resistance to three or more antibiotics, which was much higher than the percentage found among the *S. aureus* strains (20.7%).

In addition to the influence of host factors, *S. aureus* colonization is also determined by interactions with the local microbiota. It has been suggested that *S. epidermidis* in particular has the ability to directly inhibit *S. aureus* colonization*.* This is done by secreting various substances (including serine protease) that cause the release of host antimicrobial peptides. [38, 39] A recent study showed that there was a lower probability of *S. aureus* colonization in the presence of co-colonization by *Staphylococcus lugdunensis* due to the production of a “lugdunin” molecule by this bacterium, which acts as an antimicrobial agent, reducing the probability of *S. aureus* colonization. The study showed that, among 187 hospital patients, those who naturally harbored *S. lugdunensis* in the nose were six times less likely to have *S. aureus* colonization than those who did not carry it [34].

In our study, 7.1% of subjects were co-colonized by MSSA and MRSE. This is similar to the prevalence observed in a 2015 study, which found that co-colonization (with *S. aureus* and mupirocin-resistant CoNS) occurred at a rate of 6% [35]. We only observed one case of co-colonization by MRSA and MRSE (0.4%) in our study.

Our study explored the prevalence and characteristics of *S. aureus* and *S. epidermidis* nasal carriage in a representative sample of podiatrists in Spain. To the best of our knowledge, there are no published studies focusing on the prevalence of *S. aureus* and *S. epidermidis* colonization among podiatrists. Thus, this study’s findings contribute to our understanding of the carriage status among out-of-hospital health workers and could serve a basis for future studies. However, as our study is a cross-sectional study, we could not establish the prevalence of the different types of carriers: persistent, intermittent, and non-carriers. Thus, it is possible that the prevalence of carriage that we found could vary over time due to the presence of intermittent carriers.

## Conclusions

The prevalence of MSSA, MRSA, MRSE nasal carriage among podiatrists is low compared to other health personnel such as nurses, pediatrician, and even other health workers such as physiotherapists. This suggests that podiatry is not a risk factor for *S. aureus* and *S. epidermidis* colonization.

The risk factors for MSSA nasal colonization were being male, not using an aspiration system at work, and having a respiratory disease. Regarding MRSA, we did not find any significant risk factors. The risk factors for MRSE nasal colonization were being male, being ≥50 years old, having ≥15 years of podiatry experience, and not having any of the diseases examined in this study.

We must highlight the high percentage of mupirocin-resistant MRSE strains, as this is the antibiotic of choice for the treatment of nasopharyngeal carriers of *Staphylococcus* spp.

## Additional file

Additional file 1 Data recovery, *S. aureus* susceptibility, S.epidermidis susceptibility. (ZIP 55 kb)
